# Accurate AI-Based Characterization of Wound Size and Tissue Composition in Hard-to-Heal Wounds

**DOI:** 10.3390/jcm14165838

**Published:** 2025-08-18

**Authors:** Karl Lindborg, Matilda Karlsson, Ana Kotorri, Folke Sjöberg, Mats Fredrikson, Axel Haglind, Zacharias Sjöberg, Moustafa Elmasry

**Affiliations:** 1 Department of Hand Surgery, Plastic Surgery and Burns, Linköping University Hospital, 581 85 Linkoping, Sweden; karl.lindborg@liu.se (K.L.); matilda.karlsson@regionostergotland.se (M.K.); moustafa.elmasry@regionostergotland.se (M.E.); 2 Department of Biomedical and Clinical Sciences, Linköping University, 581 85 Linköping, Sweden; ana.kotorri@gmail.com (A.K.); mats.fredrikson@liu.se (M.F.); 3 Dermacut AB, 112 35 Stockholm, Sweden; zacharias@dermacut.se; 4 Department of Emergency Medicine, Linköping University, 581 85 Linköping, Sweden; axel.haglind@gmail.com

**Keywords:** chronic wounds, hard-to-heal wounds, imaging, informatics, artificial intelligence, machine learning, slough, necrosis

## Abstract

**Background:** Detailed assessments, documentation, and evaluation of the wound characteristics in hard-to-heal wounds are essential for optimizing and individualizing wound care. However, the remaining challenge in clinical care includes the lack of high accuracy and precision tools for automated wound size (surface area and depth assessment) and a wound bed evaluation, i.e., a qualitative and quantification assessment of slough and necrosis. **Objective/Methods:** This study evaluates the accuracy and precision of the AI-powered technique, SeeWound© 2, compared to digital planimetry for a wound surface area and a wound bed characterization (slough and necrosis) in “in vitro” models and in patients, and a probe for depth, including diabetic foot ulcers, venous ulcers, pressure ulcers, and ischemic ulcers. **Results:** The data show that accuracy and precision (SeeWound© 2) for the wound surface area, the depth, and the wound bed characterization (slough and necrosis) were accuracy 96.28% and 90.00%, (CV 5.56%), respectively (wound size); 90.75% and 89.55%, (CV 3.07%), respectively (wound depth); 80.30% (slough) and 84.73% (necrosis) and 93.51% (slough) (CV 4.15%) and 82.35% (CV 8.34%) (necrosis). The precision for the digital planimetry was 88.61% (CV 7.00%) (slough) 85.74% (CV 7.54%) (necrosis). **Conclusions:** The overall accuracy and precision of the AI model in identifying wound size and depth were close to 90%, except for the accuracy and precision for slough and necrosis, where levels around 80% were achieved when compared to digital planimetry. The findings for the wound surface area and depth assessments, together with quantification of slough and necrosis, suggest that the SeeWound© 2 model can offer significant clinical benefits by improving documentation and supporting decision-making in wound management.

## 1. Introduction

A hard-to-heal wound is usually caused by an underlying disease where trauma might or might not be involved; examples are diabetic foot-, venous leg-, and pressure ulcers. These wounds heal by secondary wound healing and are classified as hard-to-heal when healing is delayed past certain rate thresholds [1]. The prevalence of hard-to-heal wounds is growing and currently estimated at 1–2% across developed countries [2]. The costs and resources demanded to take care of these wounds have been studied in various healthcare systems. In wounds that do not require hospitalization, there are still significant costs of primary care visits, nurse working time, drugs, other interventions as well as dressing materials [3]. One study estimated the US Medicare costs for wound care to be at least $32 billion annually [4]. Preventive measures that target the etiology of hard-to-heal wounds are carried out by various disciplines of medicine, yet the prevalence is growing, and therefore so is the need for more efficient wound management strategies. To refine treatments, an individualized treatment strategy is needed, and this necessitates an advanced wound bed evaluation.

### Clinical Problem Addressed and Innovation

Health care professionals need to overall accurately assess the wound to be able to evaluate whether the healing process is adequate and implemented treatment strategies are functional. This includes identifying wound size, i.e., the wound surface area and depth, together with an adequate assessment of the wound bed characteristics, most commonly involving slough and necrosis.

The conventional clinical approach to measuring wound dimensions typically involves the utilization of disposable plastic rulers for surface area assessment. The evaluation of wound depth is comparatively less prevalent and less documented [5,6,7,8,9]. Wound depth measurements encompasses either (a) a subjective appraisal conducted without the aid of instruments, or (b) a method wherein healthcare practitioners uses a Q-tip for approximating depth by measuring the penetration of the Q-tip into the wound with a disposable ruler. Measurements of the wound surface area and depth acquired through these methods are well known to be imprecise and subjective [10]. Accurate estimations of wound surface area and depth are central for evaluating wound progression. Due to the absence of easy and practical clinical methods for assessing especially wound depth, there is low reliability (or even absence) of measurements documented in the electronic health records (EHRs) today. For the assessment of wound bed characteristics, such as slough and necrosis, this is evaluated by ocular inspection based on the skills of the observer. This evaluation then underlies the subsequent debridement, a very important intervention for the outcome of the healing process.

This study evaluates accuracy and precision of a mobile, automated, at the point of care device, SeeWound©2, which through AI methodology provides image-based wound surface area and depth assessments, together with a quantitative wound bed slough and necrosis characterization. A comparison to current conventional techniques, such as digital planimetry and best practices, is also undertaken.

## 2. Materials and Methods

**Figure 1:**
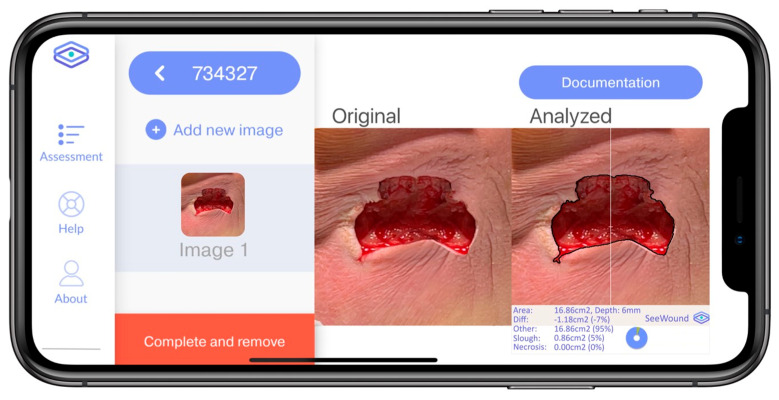
Display of a wound image in the SeeWound©2 application.

SeeWound©2 (Dermacut AB, Norr Mälarstrand, 60 11235 Stockholm, Sweden)uses a LiDAR sensor to assess wound dimensions, see Figure 1 below. The LiDAR sensor emits light that underlies the estimate of the distance between camera and wound by calculating the time it takes for light to travel from the sensor to the wound and back. In a previous publication, the influence of light interference, such as the need to use of flash, light settings in the room, and evaluation of optimal distance between the wound and the camera, has been undertaken [11]. LIDAR technology (iPad 11 Pro) and a newly developed algorithm, (SeeWound© 1(Dermacut AB, Norr Mälarstrand, 60 11235 Stockholm, Sweden)) which also incorporates an angle adjustment function that ensures control over the angular distortion during image capture, is used [11]. If the wound can be seen in the images, the previous publication shows that the wound area size can be properly depicted. Further, the paper shows that, with normal ambient light, the flash function is not needed [11]. The development of the surface area measurement algorithm has been previously described (SeeWound© 1). In short, it involved a training, validation, and a test set according to general model producing practices and includes an angle adjustment function [11]. For the wound surface estimation in the present study, the SeeWound© 1 algorithm is identical to that of SeeWound© 2. For a comparison of wound size measurements using SeeWound© 1 and 2 on the same wound and using different mobile platforms, see Table 1. The current application (SeeWound©2) is currently available for research through both TestFlight and Google Play by a written request and regulatory approval.

**Segmentation Process and Wound Depth Estimation**

**Figure 2:**
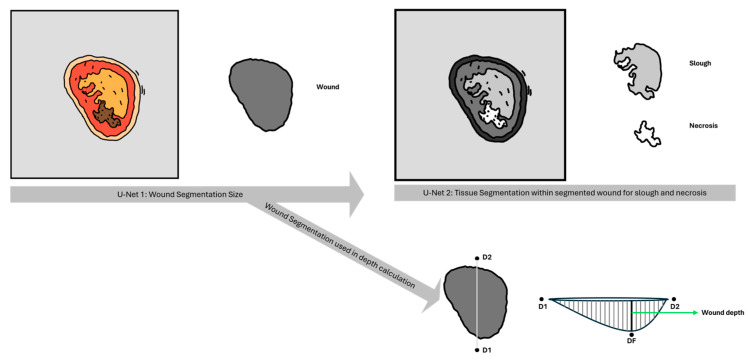
U-Net Segmentation 1 and 2 with depth calculation.

The SeeWound©2 uses two sequential convolutional neural networks based on the U-Net architecture to perform wound analysis. U-Net 1 is responsible for segmenting the wound bed. The output is subsequently used for wound area calculation, depth estimation, and serving as the input mask for the second U-Net model. U-Net 2 performs wound tissue segmentation, specifically identifying slough and necrotic tissue within the wound area previously delineated by U-Net 1. An illustration of this process is provided in Figure 2.

### 2.1. Wound Surface Area Determination

At the outpatient wound clinic of the National Burn Centre of Linköping University Hospital, wound specialists (nurses) examined the wound size in 11 patients with hard-to-heal wounds with the use of the SeeWound©2. For the accuracy estimate, wound images were captured in 11 patients (Table 2). These measurements were then compared to wound sizes obtained through manual pixel-by-pixel segmentation of a wound image performed by a wound specialist using digital planimetry, serving as the true size (digital planimetry) for the accuracy evaluation. Repeated images were also taken during the patient visit for the precision estimate in eight patients (Table 3). These paired measurements were used to assess the device’s reproducibility and precision.

### 2.2. Wound Depth Assessment

Wound depth assessment is conducted in two steps. First, the AI model is used to segment the wound area. In the second step, the boundary between healthy and wounded tissue as a reference surface is calculated, representing the estimated level of intact skin prior to injury. From this reconstructed surface, the vertical distance down to the bottom of the wound bed is measured by the LiDAR© system. This method enables estimation of wound depth by calculating the difference between the hypothetical healthy skin surface and the actual wound base (Figure 2): where D1 and D2 serve as clarification positions for the flip of the wound into portrait view, and where DF is the calculated depth between the reconstructed wound surface and the vertical distance down to actual wound bed.

**Figure 3:**
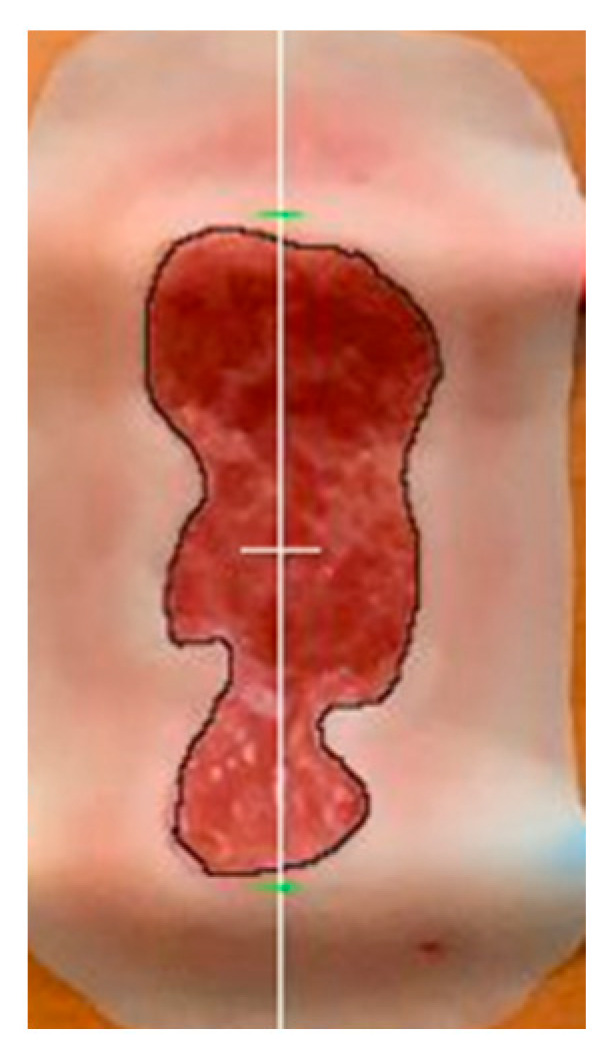
In vitro model. Artificial flat wound model used between adjustable cubes (see Figure 4).

**Figure 4:**
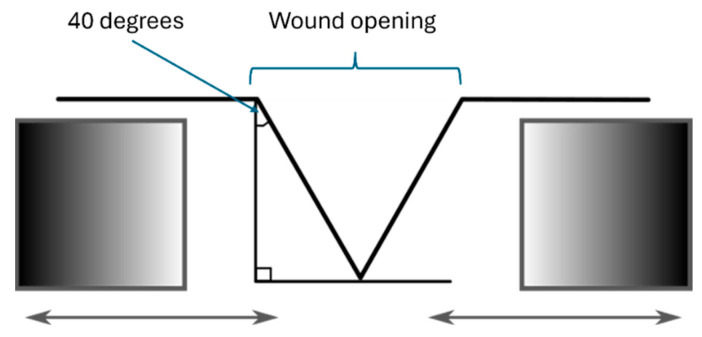
Schematic scheme of the in vitro model. Two cubes that change wound opening and wound edge angle. The flat wound model (see Figure 3) is added in the wound opening above.

The wound depth assessment functionality of SeeWound 2 was evaluated in an “in vitro” model (Figure 3 and Figure 4) (Part A) and in a clinical setting (Part B), the latter similar as described for the clinical wound surface evaluation, see above. Issues thought to affect the accuracy and precision of the wound depth estimate, such as the wound border specifics and absolute depth were examined.

The vitro model (Part A) was constructed to assess the effects of wound aperture and wound border effects on the wound depth estimate. The model includes an adjustable wound aperture in relation to the wound depth (see Figure 4). It is built on a stationary structure with two adjustable cubes, whereupon the in vitro wound model [12] (see Figure 3) is placed (in between). The structure then allowed the two adjustable objects to be moved towards each other, decreasing the wound aperture and at the same time increasing the depth of the wound. This model allows the examination of the effects of wound aperture and wound edge angle on the wound depth assessment. This is performed while the two blocks are adjusted manually and in accordance with the study protocol. It was realized early on that, given the resolution of the SeeWound© 2 depth measurement ± 3 mm, the shallowest wounds could not be differentiated from each other (see discussion section).

All in all, 51 wounds of different sizes, depth, and form were evaluated (Part A). Each wound was examined 8–10 times. The results were compared to the true depth of each (probe and ruler). Furthermore, limitations regarding technical constraints when undertaking a depth estimate were identified, and then possible constraints related to the specific dimensions of the wound (for example, wound size and depth particularly in relation to the size of the wound opening, angle of wound edges) were examined.

**Figure 5:**
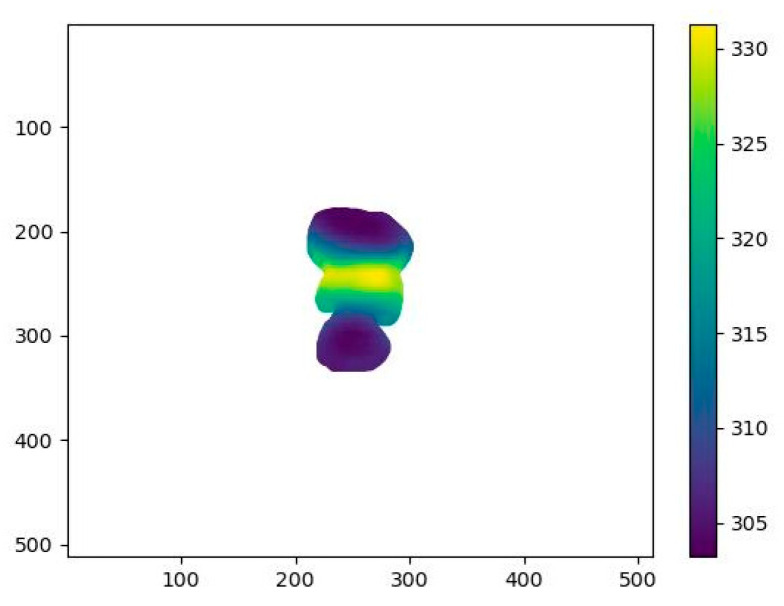
Schematic figure of detected wound contours and calculation of deepest point by SeeWound 2. Example made on in vitro model; see above for clarity.

Clinical evaluation (Part B). In the outpatient clinic of the Burn Centre of Linköping University Hospital, wound nurse specialists first measured wound depth using a sterile plastic probe and a ruler. This manual measurement was documented, after which two images of the patient’s wound were captured using the SeeWound© 2 device. The images were captured with the camera held perpendicular to the wound surface, in standard luminance patient room. SeeWound© 2 automatically detects the wound contours and calculates the deepest point based on its segmentation (see Figure 5). The automatic depth measurements from SeeWound© 2 were then compared to the manual probe measurements, which served as the ground truth (probe). Two images and measurements were performed to determine the device’s accuracy against the probe and for the reproducibility in repeated measurements.

### 2.3. Development of AI Model for the Identification of Slough and Necrosis

The U-Net architecture is well suited for identifying small objects and local differences in images, making it ideal for segmenting slough and necrosis such as in a wound bed image. As common within AI model medical applications, there are often limited data available for training, which makes the U-Net a good choice due to its ability to train efficiently on smaller datasets [13,14]. The U-Net is divided into two parts: the left side (contracting path) and the right side (expanding path). In the contracting path, the image’s height and width decrease, while the number of filters increases. This is performed through pooling operations, which reduce the spatial dimensions of the image. The goal of the contracting path is to locate and extract higher level features, such as colors, edges, and textures, through convolutions. Once the image has been reduced in size and the key features have been extracted, the expanding path performs the opposite task by upsampling the image back to its original size The expanding path uses the features identified in the contracting path (via skip connections) to recreate the full-sized image and make pixel-by-pixel predictions of the classes slough and necrosis [13,14].

**Figure 6:**
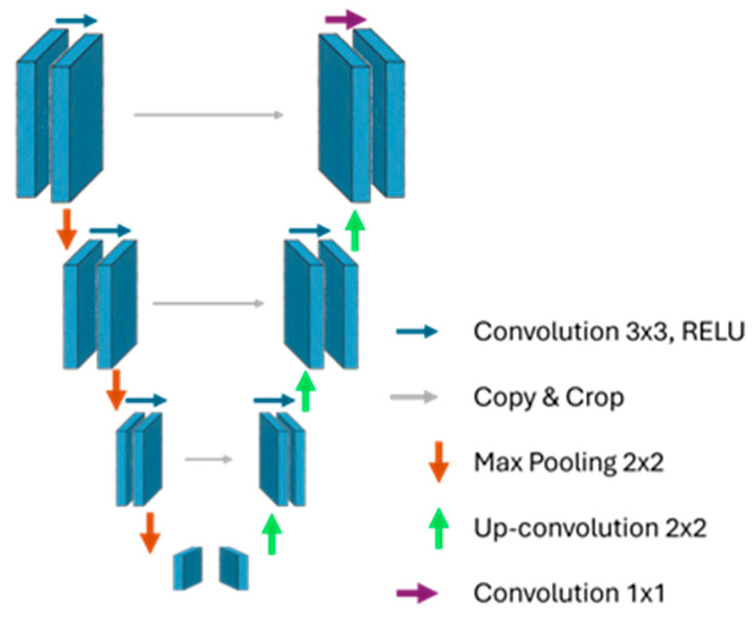
Schematic illustration of a U-net architecture.

Skip connections are links between the contracting and expanding paths, shown by the gray arrows in Figure 6 below. These connections help the network use high-resolution information from the contracting path to improve upsampling and produce a more accurate output. This prevents the model from losing spatial information during the shrinking process when rebuilding the image to its original size [13,14].

The data used for the estimate of slough and necrosis consist of images, collected from clinics using the SeeWound 1 device, of hard-to-heal wounds. In these images (*n* = 3383), the ground truths of the wound, for slough- and necrotic, segmentation were made by an experienced wound physician. Thereafter, this data set was divided into three categories: training 70%, validation 20%, and test set of 10%. The training data are entirely separated from the test set. In the training process, we used a randomization for partitioning the training data into training and validation subsets.

### 2.4. Model Training Performance on Training Validation Set for Slough and Necrosis

**Figure 7:**
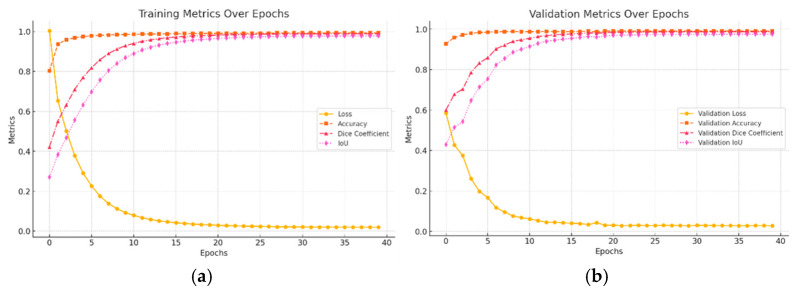
Classification of tissue, pixel by pixel on test set (training metrics over epochs) (**a**) and validation metrics over epochs (**b**).

The developed model demonstrated a consistent decrease in training loss over the epochs, which indicatively means that the model is successful in its learning of the task to segment slough and necrosis from in the wound bed. The training accuracy, dice coefficient, and intersection-over-union (iou) increase steadily throughout the model training process and reflect the model’s capability of learning to accurately classify pixels to the image’s designated classifications, and the iou reflects how good the ground truth resemblance is in the predictive and segmentation performance. Validation accuracy, dice coefficient, and the iou remained high and stable throughout the model training, indicating stable performance against the validation data set and continuous improvement in the training. See Figure 7, a and b below, for a visual representation of the training data per epoch and, respectively, for the validation performance of each epoch. The model training was stopped based on time at 10 epochs, as seen in the figure, meaning that when the model did not improve over 10 epochs, the training session was terminated to avoid over-fitting.

### 2.5. Clinical Evaluation

The final clinical evaluation of the accuracy and precision of the model was performed on two patient cohorts and included 466 and 25 patients, respectively, which were not included in the original training data. The patients included in this evaluation stem from units including vascular, dermatology, endocrinological, primary care, and municipal care. This implies a heterogeneous data set including, but not limited to, diabetic foot ulcers, venous ulcers, pressure ulcers, and ischemic ulcers. The output was evaluated by a wound care physician with ground truth segmentations (planimetry) to determine if there were correct identifications of slough and necrosis in the image. A binary comparison was also made, identifying if slough and necrosis were present or not in the wound as compared to ground truth (*n* = 466).

### 2.6. Ethics

Ethical approval was obtained from the relevant ethics committee (The National Swedish Ethics committee, Gothenburg, Dnr. 2021-04913) The ethics committee waived the need from informed consent from the patients as all the images examined did not contain any identifiable personal data and were considered de-identified/anonymized data. All patients were informed prior to image acquisition about the intended use of the images for research purposes.

### 2.7. Statistics

Data are presented as mean and standard deviation, standard error or confidence intervals. A *p* < 0.05 is considered statistically significant.

## 3. Results

### 3.1. Wound Area Measurements (SeeWound© 2) (Clinical)

Accuracy and precision for the SeeWound© 2 device were evaluated in 11 patients (see Table 1 and Table 2). Wound size ranged from 0.27 to 28.41 cm^2^ (true size). Accuracy and precision were found to be 96.53% and 92.26% (CV 5.57). In Table 2, true size and true size 2 are two measurements conducted with planimetry, and in Table 3 (see Wound 2) Measure 1 and 2 are a repeated measurement with the subject device.

### 3.2. SeeWound 2’s Wound Depth Measurements

#### Effects of Wound Opening Size and Wound Edge Angle (In Vitro)

**Figure 8:**
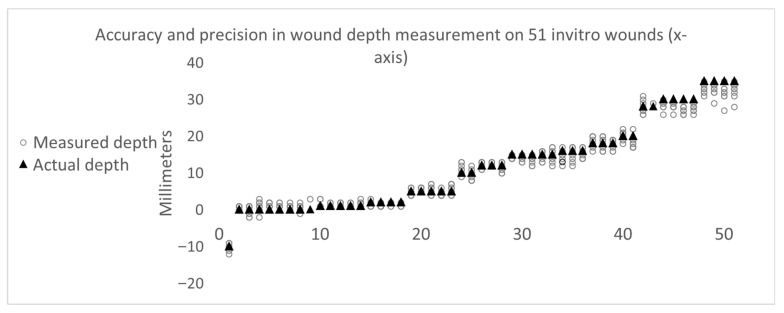
SeeWound’s accuracy and precision in depth measurements on wounds with 40 degrees or greater wound edge steepness.

SeeWound demonstrates high operability and performance when evaluated in an “in vitro” wound model with known depths, and under optimal settings, i.e., camera perpendicular to wound and on a wound with greater than 40 degrees wound edge steepness (Appendix A). For the 51 wounds (487 images) examined in the wound “in vitro” model, having depths ranging from 0 mm to 35 mm, SeeWound shows an average error from true depth of 1.13 mm (Figure 8). The largest deviation occurs when the adjustable wound module is set to have a depth of 35 mm. In this instance, SeeWound measures a depth of 27 mm, resulting in an 8 mm error (22.9% maximum error) and average precision of 88.42%. The Figure 8 below is a visual representation of all 487 measurements over the 51 wounds (*x*-axis) (Appendix B), showing the robustness of the solution and the high reproducibility and precision (measured depths circles/true depth triangle):

### 3.3. Clinical Evaluation

Wound depths were assessed in 10 patients having wounds with depths ranging from 4 mm to a maximum depth of 20 mm (measured by the probe technique), See Table 4. The corresponding wound depths registered by the SeeWound 2 device ranged from 6 to 20 mm. See Table 4. A negative depth, seen in patients 8 and 9, is where there is a hyper-granulation and therefore a growth rather than depth. SeeWound 2 measurements of +/−3 mm occur five out five times when the wound bed depth is smaller than 3 mm (see Methods section). The accuracy and precision, for depth assessment, registered for SeeWound 2 were 96.28%, and 90.00%, respectively, and the corresponding coefficient of variation was 5.56%, for the precision estimate (Table 4).

### 3.4. Artificial Intelligence Model for Tissue Segmentation

#### Classification for the Presence/Absence of Slough and/or Necrosis (Binary Outcome)

The structure of the first patient data set (466 patients including 495 images) and the performance of the model to predict the presence or absence of slough and necrosis, are shown in Table 5 below. Out of the 466 patients, 302 were found to have slough and 38 to have necrosis in the wound bed (SeeWound©2). The remaining 155 patients did have neither.

In the binary evaluation (slough/necrosis or not), the AI model correctly identifies slough in the wound bed 89.4% (accuracy) of the time, and necrosis 86.8% (accuracy) of the time. Furthermore, the AI model classifies 170 images without any slough or necrosis (actual number 155 patients). In total, the AI model correctly identifies necrotic and slough tissue in 89.12% of the patient/wound images.

Tissue segmentation (slough and necrosis) in clinical practice and compared to digital manual tracing in each wound was in a subset of the above cohort (*n* = 25).

The AI model estimation of slough and necrosis in 25 patients with wounds ranging from 0.3 to 35.42 cm^2^ in size was conducted /See Table 6). Slough and necrosis were found in ranges from 7.22 to 83.46% and 0 to 80.31%, respectively (physician planimetry). Overall accuracy and precision were accuracy 80.30% (slough) and 84.73% (necrosis); precision 93.51% (slough) (CV 4.15%) and 82.35% (CV 8.34%) (necrosis) (Ser Table 7). The precision for the digital planimetry was 88.61% (CV 7.00%) (slough) 85.74% (CV 7.54%) (necrosis).

## 4. Discussion

### 4.1. Summary of Findings

This study aimed to evaluate the accuracy and precision of the AI-powered tool SeeWound©2, for assessing the wound surface area, the wound depth, and the wound bed characteristics (slough and necrosis). The results show that SeeWound© 2, which is currently applying for a MDR certification, demonstrated robust performance metrics: for the wound surface area, the accuracy was 96.28% and the precision was 90.00%, with a coefficient of variation (CV) of 5.56%. For the wound depth, the accuracy was 90.75% and the precision was 89.55%, with a CV of 3.07%. For the wound bed characteristics, the AI model achieved accuracy values of 80.30% for slough and 84.73% for necrosis, with corresponding precision values of 88.61% (slough) and 85.74% (necrosis). These results demonstrate that SeeWound© 2 is a useful tool for wound assessment, documentation, and clinical decision support, particularly for wound size and characterization. Furthermore, comparisons with traditional methods, such as digital planimetry, indicate that SeeWound© 2 can be used as a decision support for wound size and characterization in clinical practice. The SeeWound© 2 device has an automatic export of the wound metrics to present to the major Swedish Clinical documentation system (COSMIC) by a facilitated capture algorithm, which also can be modified as needed to other specific clinical documentation systems.

### 4.2. Wound Surface Area

Among the parameters evaluated, the accuracy and precision for estimating the wound surface area were the highest. This was expected, given that the technical complexity of surface area estimation is relatively lower compared to the depth and the wound bed characteristics. The algorithm underlying the surface area model was developed in 2019 and has undergone continuous refinement, including adjustments to the camera-to-wound angle, significantly improving precision. This model, which is consistent across different mobile devices (see Table 1 above), produces results comparable to traditional planimetry. A comparison with the ASURA AI model [15] showed that SeeWound© 2 outperforms conventional ruler-based methods, which often overestimate the wound area due to simplified length × width calculations, with error margins ranging from 12% to 87% [8,16,17,18,19]. SeeWound© 2 achieved an accuracy of 96% and precision of 90%, surpassing the performance of both ASURA and traditional methods. Notably, ASURA’s model demonstrated an accuracy of 87.9% when measuring objects of known size [15], yet SeeWound© 2 was able to outperform these models in wound surface area estimations, confirming its high accuracy and precision.

### 4.3. Wound Depth

Assessing wound depth presents a greater challenge than surface measurements, primarily due to the inherent complexity of wound structures. Although high accuracy has been achieved with other systems, such as the WoundVue system (with 12.9% precision) and the method proposed by Virginia et al. (with 2% accuracy), these systems require additional hardware [20,21]. In contrast, SeeWound© 2 leverages LiDAR technology in modern smartphones, which eliminates the need for external equipment and simplifies its use in clinical settings. However, certain limitations remain. The LiDAR sensor’s depth map resolution (192 × 256 pixels) has limitations when capturing small depth variations, particularly for shallow wounds with depths under 5 mm. Additionally, the performance of the system weakens when the wound edges are steep or when the wound opening is less than 40 degrees. This is due to the light from the LiDAR sensor having difficulty reaching and reflecting on the respective wound surface. Despite these limitations, the system remains a valuable tool in clinical practice where precise depth measurements for very shallow wounds hold limited clinical value.

A review by Lasschuit et al. [10] examined available devices and methods for measuring wound depth, concluding that, despite the availability of several 3D imaging systems, depth measurements are still most reliably obtained using a sterile probe. This underscores the challenge in accurately assessing wound depth, as manual methods, such as ruler-based estimates, have demonstrated significant inaccuracies, with overestimations ranging from 12% to 87% [16,17,18,19], and inter-rater variability of estimating the wound size from −44% up to +75% [7]. These limitations are particularly pronounced in complex wound geometries or cases involving undermining [10].

### 4.4. Wound Characteristics (Slough and Necrosis)

Assessing wound bed characteristics such as slough and necrosis is particularly challenging due to the subjective nature of tissue identification, especially at the borders between tissue types. In binary evaluations, the AI model demonstrated high accuracy and precision in identifying the presence or absence of slough and necrosis. However, pixel-by-pixel evaluations of slough and necrosis were less accurate, with slough accuracy at 80.30% and necrosis accuracy at 84.73%. These results are consistent with the limitations inherent in the task of defining slough and necrosis, especially at their borders, where observer subjectivity plays a significant role. The precision for digital planimetry in slough and necrosis was 88.61% for slough and 85.74% for necrosis, with relatively high CV values (7.00% for slough and 7.54% for necrosis), suggesting that inter- and intra-rater variability, rather than the AI algorithm, is largely responsible for discrepancies in accuracy.

Mukherjee et al. [22] trained a Support Vector Machine (SVM) model using clinician-annotated images as the ground truth during the training phase. The model achieved an overall tissue segmentation accuracy of 86.13% across granulation, slough, and necrosis classes. Similarly, Morgado et al. [23] utilized a DeepLabV3-R50 segmentation model to classify wound tissue types, achieving mean absolute errors of 14.31% for slough and 8.84% for eschar. Zoppo et al. [24] evaluated several wound assessment tools and reported mean relative errors of approximately 14% for WoundViewer, Silhouette, and Visitrack, while the MOWA system exhibited a higher relative error of 23%. Fauzi et al. [25] evaluated a color-based segmentation approach using hue, saturation, and value (HSV) color space, reporting an overall accuracy of 75%, with class-specific accuracies of 63% for slough and 75.1% for eschar. They also reported inter-expert agreement scores of 67.4% and 84.3%, respectively. These studies demonstrate the ongoing challenges and variability in accurately segmenting wound tissue types, especially for slough and necrosis, underscoring the potential of AI in reducing inter-expert variability and improving wound assessment.

Interestingly, a study by Mohammed et al. [26] found that clinicians achieved 84% and 87% agreement for the presence of slough and necrosis, respectively, in nine wound images. This finding aligns with the current study, where the AI model showed a higher degree of consistency compared to clinician-based estimates. Further comparative studies by Ramachandram et al. [27] and Mukherjee et al. [22] also reported high variability among clinicians in quantifying tissue proportions, with Ramachandram et al.’s deep learning model achieving a mean intersection over union (IoU) of 86.44% for wound segmentation and 71.92% for tissue segmentation across a dataset of 58 wound images. Similarly, SeeWound© 2 demonstrated better precision and accuracy than digital planimetry, outperforming manual tracing and segmentations, which have average intra-rater ICCs of 81.02% and 78.16% for slough and necrosis respectively [27].

In clinical practice, ocular methods for estimating slough and necrosis, such as the quarter-based percentage estimation method, result in significant estimation errors. A typical absolute error in tissue estimations is ±12.5%, based on the 25% range used for proportion estimates of slough and necrosis (Bates–Jensen Wound Assessment Tool, BWAT) [28]. In contrast, SeeWound© 2 provides a more objective and consistent method for classifying tissue types, outperforming ocular assessments, which exhibit poor inter-rater agreement (ICC of 0.37–0.38) [26].

### 4.5. Limitations

#### 4.5.1. Wound Surface Area

The primary challenge is that traditional methods, such as ruler-based techniques, often lead to overestimations, with error margins ranging from 12% to 87% and inter-variations between −44% to +75% on the same wounds [7,8,16,17,18], particularly when estimating wound areas with simple length × width calculations. However, these issues are mitigated with SeeWound©2, which provides accurate estimations without the limitations associated with traditional methods.

#### 4.5.2. Wound Depth

**Figure 9:**
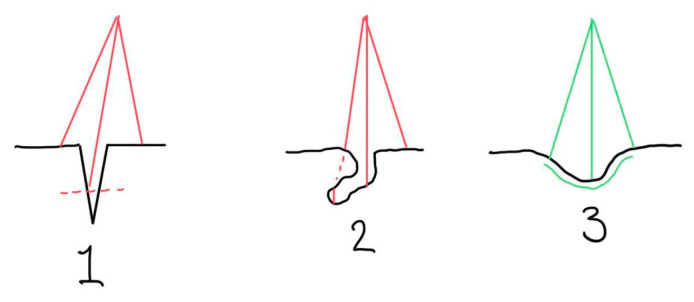
Conceptual wound edge challenges, for wound depth determinations, i.e., hidden wound parts and narrow wound edges (schematic drawing).

The most significant and obvious limitation is when parts of the wound cavity are not visible to the Lidar camera. Another limitation for wound depth measurement lies in the resolution of the LiDAR depth map and the angle of image capture. The latter is due to the image depth map resolution provided by the LiDAR technology presently uses 192 × 256 pixels. Combined with its sensitivity to the distance between the camera and the object—requiring more than 30 cm for optimal performance—this results in a limited ability to accurately capture steep edges. Even when the depth map is morphed to a higher resolution (e.g., 512 × 683 pixels), the system still challenges the sensitivity needed to detect small surface variations or subtle depth differences. Additionally, steep wound edges and non-visible wound portions in undermined or deeply cavernous wounds result in underestimated depth measurements. The light from the LiDAR sensor has difficulty reaching and reflecting on such wounds, reducing its performance in these clinical scenarios (see Figure 9 for visual explanation of the challenges). Future work to facilitate a 3-D wound bed reconstruction is currently being planned.

#### 4.5.3. Wound Bed Characteristics

Evaluating wound bed characteristics such as slough and necrosis remain challenging due to the subjective nature of tissue identification, especially at the borders between tissue types. However, SeeWound© 2 has demonstrated better consistency and higher accuracy than manual, ocular assessments, which are subject to significant observer variability. Furthermore, studies comparing AI-based segmentation with clinician-annotated images have shown that the AI model outperforms clinicians in both consistency and accuracy, highlighting the potential of AI-powered tools in wound care. We therefore suggest that the present accuracy is sufficient for clinical use. This, however, needs to be further documented in future studies.

## 5. Conclusions

The results of this study confirm that SeeWound© 2 is a highly accurate and precise tool for assessing the wound surface area, the depth, and the wound bed characteristics. The use of AI and LiDAR technology enables reliable and reproducible measurements without requiring additional external hardware, making it a practical and scalable solution for clinical practice. While there are limitations, particularly in measuring shallow and deeply undermined wounds, SeeWound© 2 outperforms traditional methods, providing clinicians with a more objective and consistent tool for wound assessment. Future advancements in algorithm training and sensor technology are expected to further improve the clinical utility of SeeWound© 2.

## Figures and Tables

**Table 1:** Measurements/device, divided equally on SeeWound 1 and 2.

Device	Mean Size (cm^2^)	Standard Deviation	Delta from Total Average	Mean Size SeeWound©1 (cm^2^)	SeeWound©1 Std (cm^2^)	Mean Size SeeWound©2 (cm^2^)	SeeWound©2 Std (cm^2^)
**Samsung Galaxy Note 9**	17.05	0.235324117	98.9%	17.00	0.20	17.10	0.26
**Samsung Galaxy Xcover 4S**	17.15	0.307064098	99.5%	16.95	0.24	17.36	0.22
**Samsung Galaxy A14**	17.46	0.253832149	101.2%	17.40	0.28	17.51	0.21
**iPhone 12 Pro**	17.53	0.235730015	101.6%	17.51	0.32	17.54	0.31
**iPhone 13 Pro**	17.12	0.318174543	99.3%	17.16	0.33	17.07	0.25
**iPad Pro (2gen)**	17.16	0.281355576	99.5%	17.08	0.23	17.25	0.30

Std: Standard Deviationd.

**Table 2:** Accuracy assessment.

Patient	SeeWound2 (cm^2^)	True Size (cm^2^)	True Size 2 (cm^2^)	Absolute Error	Absolute Error in %
1	5.45	5.66	5.68	0.21	0.039
2	6.51	6.37	6.48	0.14	0.022
3	8.5	8.66	8.53	0.16	0.019
4	3.83	3.85	4.14	0.02	0.005
5	7.91	8.21	8.6	0.3	0.038
6	14.5	14.33	14.52	0.17	0.012
7	0.31	0.27	0.33	0.04	0.129
8	13.65	14.58	14.29	0.93	0.068
9	1.95	1.99	2.13	0.04	0.021
10	27.25	27.79	28.41	0.54	0.020
11	12.5	12.37	12.79	0.13	0.010
				**Accuracy**	**96.53%**
				Std	0.036
				Standard error of the mean	0.010819869

**Table 3:** Precision assessment.

Patient	SeeWound2 Measure 1 (cm^2^)	SeeWound2 Measure 2 (cm^2^)	Diff (cm^2^)	Reproducibility	CV
1	7.98	8.58	0.6	0.075	0.051
2	1.79	2.16	0.37	0.207	0.132
3	4.66	4.6	0.06	0.013	0.009
4	15.72	15.32	0.4	0.026	0.018
5	11.51	11.25	0.26	0.023	0.016
6	1.78	1.51	0.27	0.179	0.116
7	11.85	12.11	0.26	0.022	0.015
8	1.99	2.25	0.26	0.131	0.087
			**Precision**	**91.56%**	**5.57%**
				Std	0.078
				Standard error of the mean	0.027465

**Table 4:** Wound depth assessed by the probe and SeeWound©2 device.

Patient	SeeWound 2 Depth (mm)	SeeWound 2 Depth (mm)	CV SeeWound 2 Depth (%)	Average SeeWound 2 Depth (mm)	Probe Depth (mm)	Absolute Error (mm)]	Relative Error (%)	Diff SeeWound 2 Measurements	Precision
1	4	4	0	4	3	1	0.25	0.00	0.00
2	+/−3	+/−3	“True”	“True”	2	2	0.00	0.00	0.00
3	5	4	0.157	4.5	3	1.5	0.33	1.00	0.25
4	+/−3	+/−3	“True”	“True”	0.2	0.2	0.00	0.00	0.00
6	+/−3	+/−3	“True”	“True”	1.2	1.2	0.00	0.00	0.00
7	+/−3	+/−3	“True”	“True”	2	2	0.00	0.00	0.00
8	+/−3	+/−3	“True”	“True”	0	0	0.00	0.00	0.00
9	−5	−4	−0.157	−4.5	−4	0.5	−0.11	1.00	0.25
10	−4	−6	−0.283	−5	−4	1	−0.20	2.00	0.50
11	20	20	0	20	22	2	0.10	0.00	0.00
						**Accuracy**	**96.28%**	**Precision**	**90.00%**
								**CV**	**5.56%**

**Table 5:** Binary evaluation of slough and necrosis.

Number of Images	Tissue Type	Correct Predictions	Accuracy [%]	Total Predictions to Class
155	Other	138	89.0%	170
302	Slough	270	89.4%	287
38	Necrosis	33	86.8%	37
**Total images: 495**				

**Table 6:** Accuracy (SeeWound©2) on slough and necrosis.

	AI Prediction SeeWound 2		Digital Planimetry (Physician)		Accuracy	
Patient	Slough %	Necrosis %	Slough	Necrosis	Slough	Necrosis
1	32.15	28.99	39.17	51.14	0.82	0.57
2	59.23	18.85	70.09	20.77	0.85	0.91
3	41.28	1.43	50.95	2.69	0.81	0.53
5	66.64	0.00	83.46	0.00	0.80	1.00
7	34.94	0.00	39.68	0.00	0.88	1.00
8	52.52	0.03	46.28	8.17	0.87	0.00
9	32.34	0.00	33.60	0.00	0.96	1.00
10	58.36	16.86	62.77	27.10	0.93	0.62
11	19.48	0.62	31.96	0.00	0.61	1.00
12	39.85	52.61	14.20	66.50	0.81	0.79
13	30.03	0.00	24.07	0.00	0.75	1.00
14	38.03	32.79	36.10	42.01	0.95	0.78
15	51.67	0.35	50.81	3.14	0.98	0.11
17	20.20	21.63	28.01	46.42	0.72	0.47
18	6.07	62.16	7.22	74.70	0.84	0.83
19	31.58	7.61	43.57	11.12	0.72	0.68
20	68.39	0.00	77.79	0.00	0.88	1.00
22	60.07	15.99	76.19	15.78	0.79	0.99
24	20.47	23.21	71.73	4.51	0.29	3.15
25	47.98	41.48	17.07	80.31	0.81	0.52
				**Accuracy**	**80.30%**	**84.73%**

**Table 7:** Precision (SeeWound©2) on slough and necrosis.

Patient	AI Slough	AI Necrosis	Digital Planimetry Slough	Digital Planimetry Necrosis
1	59.23	18.85	70.09	20.77
1	58.36	16.86	62.77	27.10
1	60.07	15.99	76.19	15.78
2	70.08	0.37	80.35	3.61
2	69.56	0.45	91.10	3.15
3	66.64	0.00	83.46	0.00
3	68.08	0.00	87.73	0.00
4	32.15	28.99	39.17	51.14
4	30.55	28.57	54.35	37.73
5	10.73	0.00	23.65	0.00
5	10.72	0.00	27.49	0.00
6	33.29	0	33.6	0
6	32.34	0	32.41	0
7	39.48	31.65	36.1	42.01
7	38.03	32.79	32.88	45.94
8	51.87	0.87	50.81	3.14
8	51.67	0.35	49.89	3.38
9	4.46	64.4	7.22	74.7
9	6.07	62.16	6	67.09
10	30.19	0	55.58	0
10	27.54	0	53.74	0
11	0	0	0	0
11	0	0	0	0
12	20.44	0	35.44	0
12	18.99	0	36.36	0
13	18.61	11.29	51.64	17.26
13	20.98	8.51	46.27	23.33
**Precision:**	**93.51%**	**82.35%**	**88.61%**	**85.74%**
**Average CV:**	**4.15%**	**8.34%**	**7.00%**	**7.54%**

## Data Availability

Data are available on a reasonable request to the authors.

## Abbreviations

The following abbreviations are used in this manuscript:

Spatial dimensions	Refers to the height and width of the image or feature map.
Filters	Small matrices are used to detect specific patterns in the image (e.g., edges, textures).
Convolutions	A mathematical operation that applies filters to an image to extract features.
Feature Map	The output produced by applying filters to an image, showing where certain features are present.
Upsampling	The process of increasing the size of the image or feature map, to restore original dimensions.

## Appendix A

**Table A1:** Cavity Opening.

Image Number	Test	Actual Depth [mm]	Cavity Opening [mm]	Measured Depth [mm]
1	1	23	20	17
2	1	23	20	15
3	1	23	20	15
4	1	23	20	21
5	1	23	20	17
6	1	23	20	20
7	1	23	20	17
8	1	23	20	14
9	1	23	20	18
10	1	23	20	12
11	1	23	20	20
12	1	23	20	20
13	1	23	20	17
14	2	30	12	21
15	2	30	12	18
16	2	30	12	21
17	2	30	12	21
18	2	30	12	24
19	2	30	12	23
20	2	30	12	26
21	2	30	12	30
22	2	30	12	24
23	2	30	12	26
24	2	30	12	22
25	3	30	5	26
26	3	30	5	30
27	3	30	5	26
28	3	30	5	31
29	3	30	5	29
30	3	30	5	29
31	3	30	5	26
32	3	30	5	31
33	3	30	5	17
34	3	30	5	29
35	3	30	5	16
36	3	30	5	26
37	4	30	2	26
38	4	30	2	18
39	4	30	2	13
40	4	30	2	14
41	4	30	2	17
42	4	30	2	22
43	4	30	2	20
44	4	30	2	27
45	4	30	2	16
46	4	30	2	25

## Appendix B

**Table A2:** Performance Cross Different Depth Within Known Limitations.

Image number	Actual (mm)	Predicted (mm)	Diff (mm)
1	−10	−10	0
2	−10	−10	0
3	−10	−12	2
4	−10	−9	1
5	−10	−9	1
6	−10	−10	0
7	−10	−10	0
8	−10	−11	1
9	−10	−11	1
10	−10	−9	1
11	0	0	0
12	0	0	0
13	0	1	1
14	0	1	1
15	0	1	1
16	0	1	1
17	0	1	1
18	0	0	0
19	0	1	1
20	0	**−2**	2
21	0	−1	1
22	0	1	1
23	0	1	1
24	0	−1	1
25	0	1	1
26	0	−2	2
27	0	1	1
28	0	0	0
29	0	1	1
30	0	**−2**	2
31	0	0	0
32	0	1	1
33	0	1	1
34	0	2	2
35	0	2	2
36	0	2	2
37	0	2	2
38	0	1	1
39	0	3	3
40	0	**2**	2
41	0	1	1
42	0	1	1
43	0	1	1
44	0	1	1
45	0	2	2
46	0	2	2
47	0	2	2
48	0	1	1
49	0	1	1
50	0	**2**	2
51	0	2	2
52	0	1	1
53	0	1	1
54	0	1	1
55	0	1	1
56	0	1	1
57	0	1	1
58	0	1	1
59	0	1	1
60	0	**2**	2
61	0	0	0
62	0	0	0
63	0	1	1
64	0	1	1
65	0	1	1
66	0	1	1
67	0	1	1
68	0	1	1
69	0	1	1
70	0	**1**	1
71	0	1	1
72	0	0	0
73	0	1	1
74	0	1	1
75	0	1	1
76	0	0	0
77	0	−1	1
78	0	0	0
79	0	2	2
80	0	**3**	3
81	1	1	0
82	1	3	2
83	1	3	2
84	1	1	0
85	1	1	0
86	1	1	0
87	1	1	0
88	1	1	0
89	1	1	0
90	1	**1**	0
91	1	1	0
92	1	1	0
93	1	1	0
94	1	1	0
95	1	1	0
96	1	1	0
97	1	2	1
98	1	1	0
99	1	2	1
100	1	**1**	0
101	1	2	1
102	1	2	1
103	1	2	1
104	1	1	0
105	1	2	1
106	1	2	1
107	1	1	0
108	1	1	0
109	1	1	0
110	1	**1**	0
111	1	1	0
112	1	1	0
113	1	1	0
114	1	1	0
115	1	1	0
116	1	1	0
117	1	1	0
118	1	2	1
119	1	1	0
120	1	**2**	1
121	1	3	2
122	1	2	1
123	1	2	1
124	1	2	1
125	1	2	1
126	1	1	0
127	1	1	0
128	1	1	0
129	1	2	1
130	1	**1**	0
131	2	2	0
132	2	2	0
133	2	2	0
134	2	2	0
135	2	1	1
136	2	1	1
137	2	2	0
138	2	3	1
139	2	1	1
140	2	**1**	1
141	2	2	0
142	2	2	0
143	2	1	1
144	2	1	1
145	2	1	1
146	2	1	1
147	2	1	1
148	2	1	1
149	2	2	0
150	2	**1**	1
151	2	1	1
152	2	2	0
153	2	2	0
154	2	2	0
155	2	2	0
156	2	2	0
157	2	2	0
158	2	2	0
159	2	2	0
160	2	**2**	0
161	2	2	0
162	2	2	0
163	2	2	0
164	2	2	0
165	2	2	0
166	2	1	1
167	2	2	0
168	2	2	0
169	2	1	1
170	2	**2**	0
171	5	6	1
172	5	5	0
173	5	4	1
174	5	6	1
175	5	4	1
176	5	4	1
177	5	5	0
178	5	4	1
179	5	4	1
180	5	**5**	0
181	5	6	1
182	5	5	0
183	5	6	1
184	5	6	1
185	5	6	1
186	5	5	0
187	5	6	1
188	5	5	0
189	5	6	1
190	5	**6**	1
191	5	6	1
192	5	5	0
193	5	7	2
194	5	4	1
195	5	5	0
196	5	4	1
197	5	5	0
198	5	7	2
199	5	6	1
200	5	**6**	1
201	5	4	1
202	5	4	1
203	5	5	0
204	5	5	0
205	5	4	1
206	5	4	1
207	5	4	1
208	5	4	1
209	5	5	0
210	5	**5**	0
211	5	6	1
212	5	7	2
213	5	5	0
214	5	5	0
215	5	7	2
216	5	6	1
217	5	4	1
218	5	4	1
219	5	6	1
220	5	**7**	2
221	10	10	0
222	10	13	3
223	10	11	1
224	10	10	0
225	10	11	1
226	10	9	1
227	10	13	3
228	10	12	2
229	10	10	0
230	10	**8**	2
231	10	10	0
232	10	8	2
233	10	8	2
234	10	10	0
235	10	11	1
236	10	12	2
237	10	10	0
238	10	9	1
239	10	10	0
240	10	**8**	2
241	12	12	0
242	12	11	1
243	12	13	1
244	12	13	1
245	12	12	0
246	12	13	1
247	12	13	1
248	12	13	1
249	12	11	1
250	12	**13**	1
251	12	13	1
252	12	13	1
253	12	12	0
254	12	12	0
255	12	12	0
256	12	12	0
257	12	12	0
258	12	12	0
259	12	**11**	1
260	12	13	1
261	12	12	0
262	12	10	2
263	12	10	2
264	12	11	1
265	12	10	2
266	12	12	0
267	12	12	0
268	12	12	0
269	12	11	1
270	12	**13**	1
271	15	15	0
272	15	14	1
273	15	15	0
274	15	14	1
275	15	14	1
276	15	14	1
277	15	15	0
278	15	14	1
279	15	14	1
280	15	**14**	1
281	15	13	2
282	15	15	0
283	15	15	0
284	15	15	0
285	15	14	1
286	15	13	2
287	15	15	0
288	15	14	1
289	15	15	0
290	15	**15**	0
291	15	14	1
292	15	14	1
293	15	12	3
294	15	14	1
295	15	14	1
296	15	13	2
297	15	14	1
298	15	15	0
299	15	12	3
300	15	**13**	2
301	15	14	1
302	15	16	1
303	15	13	2
304	15	16	1
305	15	15	0
306	15	16	1
307	15	16	1
308	15	15	0
309	15	16	1
310	15	**15**	0
311	15	16	1
312	15	14	1
313	15	13	2
314	15	14	1
315	15	16	1
316	15	17	2
317	15	15	0
318	15	17	2
319	15	12	3
320	15	**15**	0
321	16	15	1
322	16	15	1
323	16	16	0
324	16	16	0
325	16	12	4
326	16	17	1
327	16	16	0
328	16	13	3
329	16	14	2
330	16	**12**	4
331	16	16	0
332	16	15	1
333	16	17	1
334	16	13	3
335	16	17	1
336	16	14	2
337	16	16	0
338	16	16	0
339	16	17	1
340	16	**14**	2
341	16	17	1
342	16	15	1
343	16	14	2
344	16	16	0
345	16	16	0
346	16	17	1
347	16	16	0
348	16	14	2
349	16	14	2
350	16	**16**	0
351	18	20	2
352	18	18	0
353	18	17	1
354	18	20	2
355	18	16	2
356	18	19	1
357	18	20	2
358	18	20	2
359	18	18	0
360	18	**20**	2
361	18	16	2
362	18	19	1
363	18	16	2
364	18	16	2
365	18	17	1
366	18	20	2
367	18	20	2
368	18	18	0
369	18	19	1
370	18	**18**	0
371	18	17	1
372	18	18	0
373	18	19	1
374	18	16	2
375	18	19	1
376	18	19	1
377	18	19	1
378	18	16	2
379	18	18	0
380	18	**19**	1
381	20	21	1
382	20	20	0
383	20	21	1
384	20	21	1
385	20	21	1
386	20	22	2
387	20	20	0
388	20	21	1
389	20	21	1
390	20	21	1
391	20	20	0
392	20	20	0
393	20	18	2
394	20	19	1
395	20	19	1
396	20	**19**	1
397	20	22	2
398	20	19	1
399	20	18	2
400	20	17	3
401	20	17	3
402	20	17	3
403	20	17	3
404	20	18	2
405	20	17	3
406	20	**17**	3
407	20	17	3
408	28	27	1
409	28	26	2
410	28	28	0
411	28	27	1
412	28	31	3
413	28	29	1
414	28	30	2
415	28	26	2
416	28	27	1
417	28	**29**	1
418	30	29	1
419	30	29	1
420	30	29	1
421	30	28	2
422	30	29	1
423	30	26	4
424	30	30	0
425	30	29	1
426	30	28	2
427	30	**29**	1
428	30	29	1
429	30	29	1
430	30	28	2
431	30	26	4
432	30	28	2
433	30	29	1
434	30	28	2
435	30	28	2
436	30	28	2
437	30	**26**	4
438	30	26	4
439	30	28	2
440	30	28	2
441	30	27	3
442	30	28	2
443	30	26	4
444	30	27	3
445	30	27	3
446	30	28	2
447	30	**28**	2
448	30	29	1
449	30	27	3
450	30	28	2
451	30	28	2
452	30	28	2
453	30	26	4
454	30	28	2
455	30	28	2
456	30	27	3
457	30	**27**	3
458	35	33	2
459	35	35	0
460	35	32	3
461	35	33	2
462	35	32	3
463	35	31	4
464	35	33	2
465	35	34	1
466	35	33	2
467	35	**32**	3
468	35	34	1
469	35	34	1
470	35	33	2
471	35	32	3
472	35	32	3
473	35	34	1
474	35	29	6
475	35	33	2
476	35	33	2
477	35	**33**	2
478	35	32	3
479	35	31	4
480	35	31	4
481	35	32	3
482	35	32	3
483	35	27	8
484	35	33	2
485	35	35	0
486	35	35	0
487	35	**34**	1
488	35	34	1
489	35	28	7
490	35	33	2
491	35	31	4
492	35	33	2
493	35	34	1
494	35	32	3
495	35	33	2
496	35	34	1
497	35	**35**	0
